# Structure of the quaternary complex of histone H3-H4 heterodimer with chaperone ASF1 and the replicative helicase subunit MCM2

**DOI:** 10.1007/s13238-015-0190-0

**Published:** 2015-07-18

**Authors:** Hong Wang, Mingzhu Wang, Na Yang, Rui-Ming Xu

**Affiliations:** National Laboratory of Biomacromolecules, Institute of Biophysics, Chinese Academy of Sciences, Beijing, 100101 China; University of Chinese Academy of Sciences, Beijing, 100049 China

**Dear Editor,**

The building block of eukaryotic chromatin is the nucleosome core particle (NCP), which is consisted of ~146 bps of DNA wrapped around an octamer of core histones. A tetramer of histone H3 and H4 and two H2A-H2B dimers form the histone octamer (Kornberg, 1974; Luger et al., 1997; Thomas and Kornberg, 1975). During DNA replication, nucleosome disassembly and reassembly occurs at the replication fork, and histone chaperons CAF-1 and ASF1 are principally responsible for the deposition of histones H3 and H4 onto replicated DNA (Kaufman et al., 1995; Tyler et al., 1999). ASF1 binds a heterodimeric histone H3-H4 complex through its conserved N-terminal domain (aa 1–155) and impedes the formation of the (H3-H4)_2_ tetramer both inside the nucleus and in the cytoplasm (English et al., 2005). Humans have two ASF1 paralogs, ASF1a and ASF1b, both of which can be co-purified with the MCM 2-7 replicative helicase and histones H3 and H4 from HeLa cell nuclear extract, while only the MCM2 subunit can be pulled down by ASF1a or ASF1b together with H3 and H4 when using cytosolic extract of the same cells (Groth et al., 2007). *In vitro* studies showed that an N-terminal of human MCM2 (aa 63–154) binds histone H3 directly through a conserved motif (Foltman et al., 2013; Ishimi et al., 1998). The crystal structure of the MCM2 fragment in complex with the tetrameric (H3-H4)_2_ complex has recently determined (Richet et al., 2015). However, it remains unknown whether MCM2 can bind histone H3 and H4 together with ASF1, and if so, whether the two bind in a synergistic manner or independently.

To answer these questions, we embarked on the characterization and structure determination of a quaternary complex of MCM2, ASF1 and histones H3 and H4. First, we find that they form an apparent 1:1:1:1 complex. The N-terminal domain of human MCM2 (aa 63–154), the globular domain of ASF1a (aa 1–157) and full-length human histones H3 and H4 were expressed in *E. coli*. A stable complex of the four proteins was obtained at a salt concentration of 0.5 mol/L NaCl (Fig. 1A). The tetrameric complex eluded from a Superdex 200 10/300 GL size exclusion column (GE Healthcare) at an elution volume of 14.38 mL (Fig. S1), corresponding to an apparent molecular weight of about 60 kDa, which is compatible with a stoichiometry of 1:1:1:1 of the four components. We then crystallized and solved a 3.5-Å structure of the quaternary complex, and the structure was solved by molecular replacement (see Supplemental Material for details). There are six MCM2-ASF1-H3-H4 tetramers in one asymmetric unit, the inter-tetramer contacts appear to be non-physiological, and we will concentrate our analyses on one tetramer henceforth.

**Figure 1:**
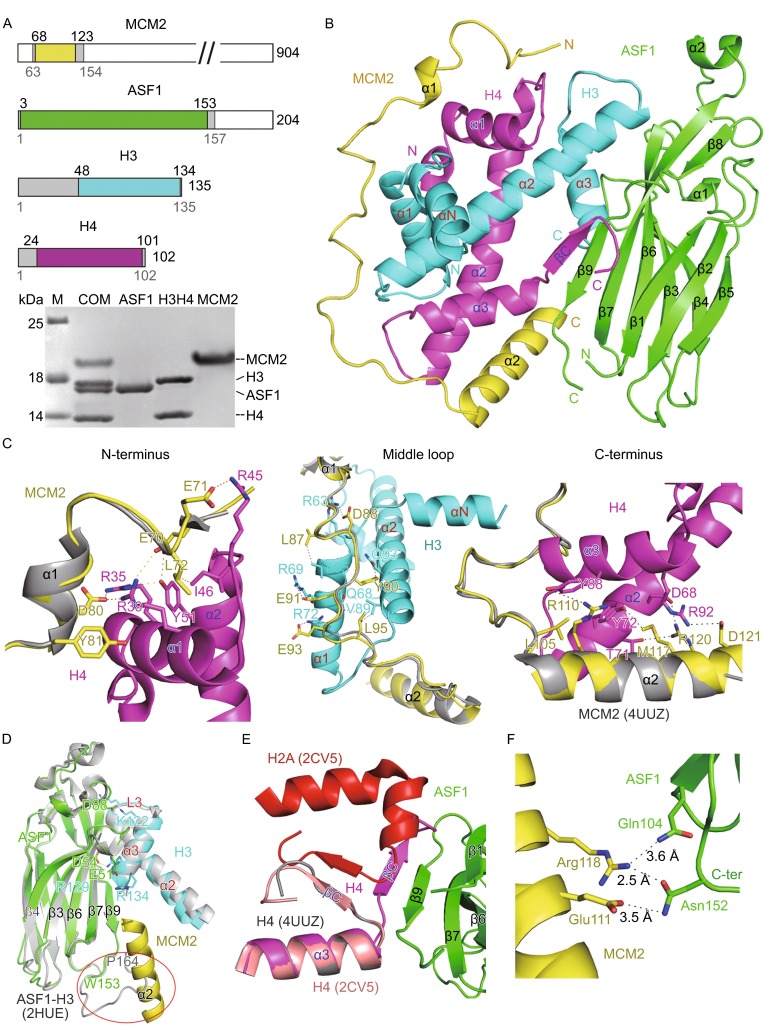
**Structure of the MCM2-ASF1-H3-H4 complex**. (A) Schematic diagram showing the truncated fragments of the four proteins. MCM2, ASF1, H3 and H4 are shown in yellow, green, cyan and magenta respectively (color coded the same in all figures). Disordered regions in the structure are shown in gray. An image of the coomassie-stained SDS-PAGE gel of the purified complex showing apparent stoichiometry of the four proteins. MCM2 appears to have an anomalous SDS-PAGE migration profile, as a Mass spectrometric measurement indicates a molecular weight 10,774 Da (data not shown). (B) A ribbon diagram showing the overall structure of the quaternary complex. (C) Interactions between MCM2 and H3-H4 heterodimer. The structure of MCM2 from the ternary MCM2-H3-H4 structure (PDB code: 4UUZ, shown in gray) is superimposed for comparison. Three panels indicate N-terminal, middle and C-terminal binding regions of MCM2. Residues involved in intermolecular interactions are shown in a stick model (carbon, yellow, magenta and cyan; nitrogen, blue; oxygen, red). Dashed lines indicate intermolecular hydrogen bonds. (D) Interactions between ASF1 and histone H3. Superposition of ASF1 and H3 from our quaternary structure and that from the ternary ASF1-H3-H4 structure (PDB code: 2HUE, shown in gray). C-terminal ends of ASF1 in the two structures and α2 MCM2 are highlighted inside the red circle. (E) Interactions between ASF1 and histone H4. The βC strand of H4 in the MCM2-H3-H4 ternary structure and in a human nucleosome structure (shown in gray and magenta, respectively) are aligned with that in the quaternary structure. Histone H2A in the nucleosome structure are shown in red. (F) Direct interactions between MCM2 and ASF1

The H3-H4 heterodimer is centrally located in the quaternary complex, enclosed on one side by MCM2 and the opposite side by ASF1 (Fig. 1B). MCM2 is composed of an N-terminal tail followed by a short helix (α1), which is connected to a C-terminal helix (α2) via a long loop. The extensive interaction between MCM2 and H3-H4 buries a total surface area of 1918 Å^2^. The long loop snakes through the convex surface of the H3-H4 dimer, accounting for ~2/3 of the interaction surface area with H3-H4. A comparison with the recently published structure of the (MCM2-H3-H4)_2_ complex (PDB code: 4UUZ) reveals that MCM2 interacts with H3-H4 similarly in the two complexes (Fig. 1C) (Richet et al., 2015). The observed interactions between ASF1 and H3-H4, mainly those between the globular domain of ASF1 and the C-terminal α3 helix and the loop region between α2 and α3 of histone H3, also conform to those observed in the ternary ASF1-H3-H4 complex (PDB code: 2HUE) (English et al., 2006; Natsume et al., 2007). Interesting differences occur at the C-terminal end of ASF1 where 11 residues (aa 154–164) are disordered in our structure (Fig. 1D). This segment of ASF1 would bump into α2 of MCM2 if it took the conformation of that in the ternary ASF1-H3-H4 complex. The projection of the C-terminal segment of ASF1 is largely determined by the position of the immediately N-terminal strand β9, which is stabilized by antiparallel pairing with the C-terminal β-strand (βC) of histone H4 (Fig. 1B). It is interesting to note that while βC is positioned similarly in our quaternary complex and the ASF1-H3-H4 ternary complex, it contrasts sharply with that found in the (MCM2-H3-H4)_2_ complex (Fig. 1E). The conformation of βC in the latter complex (PDB code: 4UUE, shows in gray) is similar to that of the nucleosomal H4, although not being stabilized by pairing with the C-terminal β strand of histone H2A as in NCP (PDB code: 2CV5, H4 in pink and H2A in red) (Fig. 1E). Finally, although scarce, direct interactions between MCM2 and ASF1 are observed in the quaternary complex: the guanidine group of Arg118 of MCM2 forms a hydrogen bond with the hydroxyl group of Asn152 of ASF1; and weaker interactions between Gln104 and Asn152 of ASF1 and Arg118 and Glu111 of MCM2, respectively (Fig. 1F).

The principal functions of histone chaperones are to keep the histones in their pre-depositional, DNA-free state. Superposition of our structure with the NCP structure (PDB code: 2CV5, shows in gray) via the H3-H4 heterodimer shows that (1) ASF1 occupies a position blocking the dimerization interface of histone H3, thus preventing the formation of a (H3-H4)_2_ tetramer (Fig. 2A, Region 1); (2) MCM2 occludes the binding of histone H2B to histone H4 through its α2 helix, which impedes the association of H2A-H2B heterodimers with (H3-H4)_2_ tetramer to form an octamer (Fig. 2A, Region 2); (3) both the binding of MCM2 to the positively charged surface of the H3-H4 heterodimer and the protrusion of the C-terminal α2 helix of ASF1 would obstruct the wrapping of DNA in NCP. These features of MCM2 and ASF1 define them as *bona fide* histone H3-H4 chaperones.

**Figure 2:**
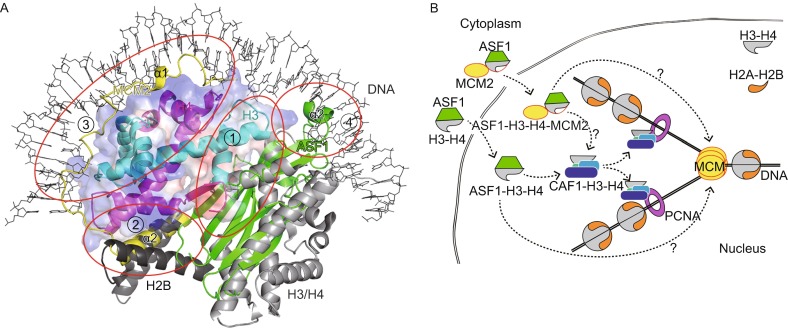
**Possible biological functions of the MCM2-ASF1-H3-H4 complex**. (A) The binding of MCM2 and ASF1 obstructs the formation of NCP and a histone (H3-H4)_2_ tetramer. A human nucleosome structure (PDB code: 2CV5, shown in gray) is aligned with the MCM2-ASF1-H3-H4 structure via the H3-H4 heterodimer. Four obstructed regions are enclosed in red circles and numbered according to the order in which they were referenced in the text. (B) A model of possible biological functions of the MCM2-ASF1-H3-H4 quaternary complex in the cytoplasm and in the nucleus. The cytosolic MCM2 may facilitate nuclear import of histones H3 and H4. In the nucleus, ASF1 “hands” the H3-H4 heterodimer to the CAF-1 complex prior to the deposition onto replicated DNA, in the presence or absence of MCM2. The ASF1-H3-H4 complex might also be directly recruited to the replication fork by interaction with the MCM complex

Our structural study revealed the structural basis for the interaction between the ASF1-bound H3-H4 complex with MCM2. During replication-dependent or repair-coupled chromatin assembly, it is generally believed that ASF1 “hands” the H3-H4 heterodimer to the CAF-1 chaperonin complex prior to the deposition onto replicated DNA. Exactly how ASF1 is recruited to the replication or damage foci remains poorly understood, except that ASF1 is known to directly interact with the CAF-1 complex (Mello et al., 2002). A recent result also showed the association of MCM complex with ASF1 in U2OS cells (Drissi et al., 2015). The interaction between MCM2 and the ASF1-H3-H4 complex may function together with CAF-1 for efficient recruitment of the ASF1-H3-H4 complex, or alternatively, serves as an additional recruitment mechanism. As mentioned earlier, MCM2 co-purifies with ASF1 and histones H3 and H4 in the absence of other helicase components in the cytoplasm (Groth et al., 2007). Therefore, MCM2 appears to have a chaperoning function outside the context of a helicase complex, which functions exclusively in the nucleus. As with some other histone chaperones, such as NASP (Campos et al., 2010), the cytosolic MCM2 may also facilitate the nuclear import of histones H3 and H4. A summary of possible cellular functions of the interactions among MCM2, ASF1 and histones H3 and H4 is depicted in Fig. 2B, and the structural basis for their interactions learned here should help the dissection of their functions in chromatin biology.

The coordinates and structure factors of the MCM2-ASF1-H3-H4 quaternary complex have been deposited in PDB under the accession code 5C3I.

## FOOTNOTES

We thank SSRF beamline scientists for help with data collection and Qianglin Fang for participation at an early stage of the work. We also thank grant supports from the National Natural Science Foundation of China (Grant Nos. 31210103914 and 31430018 to R.M.X.), the National Basic Research Program (973 Program) (No. 2015CB856202 to N.Y.), the Strategic Priority Research Program (XDB08010100) and the Key Research Program (KJZD-EW-L05) of Chinese Academy of Sciences (CAS). N.Y. is also supported by the Youth Innovation Promotion Association of CAS.

All authors declare that they have no conflict of interest. This article does not contain any studies with human or animal subjects performed by any of the authors.

## Electronic supplementary material

Supplementary material 1 (PDF 249 kb)
