# Supplementary material for: Structure of the quaternary complex of histone H3-H4 heterodimer with chaperone ASF1 and the replicative helicase subunit MCM2
Source: Protein Cell. 2015 Jul 18;6(9):693–7. doi: 10.1007/s13238-015-0190-0 (PMC4537477; doi:10.1007/s13238-015-0190-0)
Supplement: Supplementary file 1 — Supplementary material 1 (PDF 249 kb) [file 13238_2015_190_MOESM1_ESM.pdf]

Supplemental Materials

cDNA fragments encoding human MCM2 (aa 63-154) and ASF1a (aa 1-157) were cloned into a pET-28a-SUMO and a pRSFDuet-1 vector (Novagen), respectively. Both proteins were expressed in the BL21 CodonPlus (DE3) RIL strain of *E. coli* as fusion proteins with an N-terminal poly(His)-SUMO or C-terminal poly(His) tag. Protein expressions were induced with 0.25 mM β-D-1-thiogalactopyranoside (IPTG) at 16 ℃ overnight when cell density reached OD_{600} ~0.8. Cells were harvested, resuspended in buffer A (20 mM Tris-HCl, pH 8.0, 500 mM NaCl, 20 mM imidazole and 1mM PMSF) and lysed by sonication. Cell lysate was separated by centrifugation and the supernatant was loaded onto a Ni-IDA column (GE Healthcare) and the target protein was eluted by buffer B (20 mM Tris-HCl, pH 8.0, 100 mM NaCl, 250 mM imidazole). Eluted fractions were pooled and incubated with SUMO protease at 4℃ for 16 hours to cut the His-SUMO tag off the MCM2 fusion protein. The cleaved tag was removed by chromatography using a Hitrap Q column (GE Healthcare) with a linear gradient of 0.1-1.0 M NaCl. The same step was used to purify ASF1-His. Full-length human histones H3.1 and H4 were co-expressed using a bicistronic plasmid using a pRSFDuet-1 vector, and the H3-H4 complex was purified under native conditions as previously described (Liu et al., 2012).

The purified proteins, MCM2, ASF1 and the histone H3-H4 complex were mixed at an approximate molar ratio of 1.5:1.5:1 and dialyzed against buffer C (20 mM Tris-HCl, pH 8.0, 500 mM NaCl, 1 mM EDTA, 1 mM DTT) at 4℃ for 4 hours. Then, the sample was concentrated and applied to a Superdex HiLoad 200 16/60 column (GE Healthcare) pre-equilibrated with buffer C. The eluted fractions were analyzed by SDS-PAGE, and the target quaternary complex was pooled and concentrated to ~25 mg/ml for crystallization. Crystallization was carried out at 16℃ by the hanging drop vapor diffusion method. Crystals were obtained with the mixture of 1.0 μl protein solution and 1.0 μl reservoir solution in a condition containing 0.1 M Tris-HCl pH 8.0, 20% PEG3350 and 2% Tacsimate. For cryogenic data collection, crystals were flash-frozen in liquid nitrogen using a cryo-protectant solution prepared from the reservoir solution supplemented with 20% Glycerol. Diffraction data were collected at beamline BL17U of Shanghai Synchrotron Radiation Facility (SSRF) using a Quantum 315r CCD detector (ADSC). A 3.5Å native data set was collected at a wavelength of 0.9792 Å and processed using HKL2000 software (Otwinowski and Minor, 1997).

The crystal belongs to the $P3_121$ space group, and there are six MCM2-ASF1-H3-H4-tetramers in one asymmetric unit. The structure was solved by molecular replacement (MR) using PHASER software in the CCP4 program suite (McCoy, 2007), using the ternary structure of human ASF1-H3-H4 as the search model (PDB code 2IO5). The electron density map was of high quality after several rounds of refinement with REFMAC (Murshudov et al., 1997; Natsume et al., 2007). Model of MCM2 molecule was built by docking the human MCM2-H3-H4 structure (PDB code 4UUZ) to the MR model by superposition of H3-H4 heterodimers in the two structures (Fig. S2). The structure was further refined with REFMAC and COOT (Emsley and Cowtan, 2004), and the refined model has R_{work} and R_{free} values of 0.230 and 0.275, respectively. Further data analyses using PHENIX (Adams et al., 2010) indicated merohedral twinning with a twinning fraction of 0.21. Refinement with intensity-based twin correction using REFMAC, the R_{work} and R_{free} of the final model are 0.216 and 0.253, respectively. Detailed statistics for data collection and refinement are shown in Table S1.

Table S1. Statistics for crystallographic analysis

Data collection	
Wave length (Å)	0.9792
Space group	$P3_121$
Cell dimensions	
a, b, c (Å)	147.7, 147.7, 261.8
α, β, γ (°)	90, 90, 120
Resolution (Å)[a]	50-3.50 (3.63-3.50)
R_{merge}[b]	0.161 (0.690)
I/σI	12.8 (2.8)
Completeness (%)	99.8 (100.0)
Total/Unique reflections	388727/42834
Redundancy	9.1 (9.2)
Refinement Statistics	
Rwork/Rfree[c]	0.216/0.253
No. atoms	
Protein	17655
B factor ($Å^2$)	
Protein	47.4
rmsd bonds (Å)	0.005
rmsd angles (°)	0.915
Ramachandran plots	
favored	98.4%
allowed	1.6%
outlier	0.0%

[a] Values in the parentheses are that for the highest resolution range.
[b] R_{merge}= |I - <I>|/<I>, where I and <I> are the averaged intensity of multiple measurements of the same reflection. The summation is over all the observed reflections.
[c] R-factor = $||F_o| - |F_c||/|F_o|$, where F_o denotes the observed structure factor amplitude and F_c denotes the structure factor calculated from the model. R-work was calculated using the diffraction data used throughout the refinement, and R-free was calculated using the 5% of the data set aside during refinement.

Supplemental Figure S1

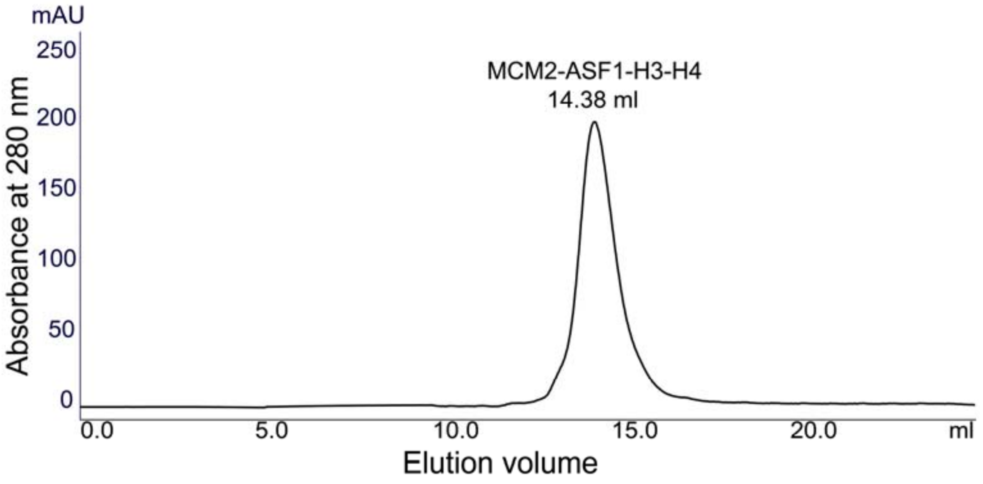

Fig. S1 Elution profile of the MCM2-ASF1-H3-H4 quaternary complex from a Superdex 200 10/300 GL size exclusion column (GE Healthcare).

Supplemental Figure S2

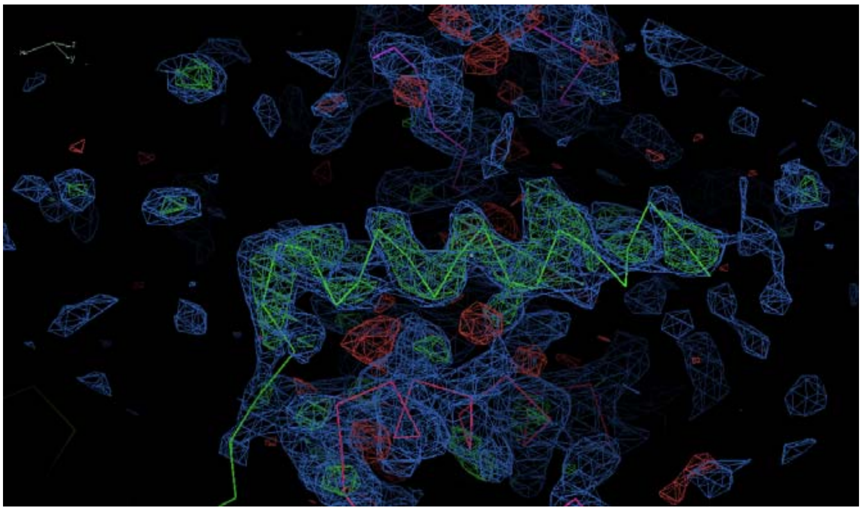

Fig. S2 Electron density map of the MCM2-ASF1-H3-H4 structure at 3.5 Å resolution. 2Fo-Fc map at 1.0 sigma (blue color) and Fo-Fc map at 2.5 sigma (green color) of the α2 helix of MCM2.

Supplemental References

Adams, P.D., Afonine, P.V., Bunkoczi, G., Chen, V.B., Davis, I.W., Echols, N., Headd, J.J., Hung, L.W., Kapral, G.J., Grosse-Kunstleve, R.W., *et al.* (2010). PHENIX: a comprehensive Python-based system for macromolecular structure solution. Acta crystallographica Section D, Biological crystallography 66, 213-221.

Emsley, P., and Cowtan, K. (2004). Coot: model-building tools for molecular graphics. Acta crystallographica Section D, Biological crystallography 60, 2126-2132.

Liu, C.P., Xiong, C., Wang, M., Yu, Z., Yang, N., Chen, P., Zhang, Z., Li, G., and Xu, R.M. (2012). Structure of the variant histone H3.3-H4 heterodimer in complex with its chaperone DAXX. Nature structural & molecular biology 19, 1287-1292.

McCoy, A.J. (2007). Solving structures of protein complexes by molecular replacement with Phaser. Acta crystallographica Section D, Biological crystallography 63, 32-41.

Murshudov, G.N., Vagin, A.A., and Dodson, E.J. (1997). Refinement of macromolecular structures by the maximum-likelihood method. Acta crystallographica Section D, Biological crystallography 53, 240-255.

Otwinowski, Z., and Minor, W. (1997). Processing of X-ray diffraction data collected in oscillation mode. Method Enzymol 276, 307-326.
